# Serum leptin, resistin, and adiponectin levels in obese and non-obese patients with newly diagnosed type 2 diabetes mellitus: A population-based study

**DOI:** 10.1097/MD.0000000000019052

**Published:** 2020-02-07

**Authors:** Wei Liu, Xianghai Zhou, Yufeng Li, Simin Zhang, Xiaoling Cai, Rui Zhang, Siqian Gong, Xueyao Han, Linong Ji

**Affiliations:** a Department of Endocrinology and Metabolism, Peking University People's Hospital; b Department of Endocrinology and Metabolism, Pinggu Hospital, Beijing, China.

**Keywords:** adiponectin, adiponectin-to-leptin ratio, leptin, resistin, type 2 diabetes

## Abstract

Disturbances in adipocytokine profiles can contribute to peripheral insulin resistance and impairment of insulin production, which are 2 primary pathophysiological mechanisms involved in type 2 diabetes mellitus (T2DM). Previous studies of disturbed adipocytokine profiles have resulted in ambiguous findings; therefore, we conducted the current study comparing leptin, resistin, and adiponectin concentrations in patients with newly diagnosed T2DM who had normal body mass index (BMI) and those who were obese.

We studied a population-based cohort of healthy participants and those with newly diagnosed T2DM. A normal BMI group was randomly selected; age- and sex-matched obese participants were recruited. Circulating leptin, resistin, and adiponectin concentrations were measured and compared between groups using analysis of variance; binary logistic regression analysis was then performed to compare the normal BMI and obese groups.

In total, 85 healthy participants and 38 patients with diabetes (19 with normal BMI and 17 who were obese) were enrolled. After adjustment for BMI and waist circumference, the median leptin concentration was higher in the obese group (6.77 (3.89–10.73) ng/mL) than in the normal BMI group (1.69 (0.80–3.89) ng/mL) (*P* = .007), whereas the median adiponectin concentration was lower in the obese group (1.03 (0.75–2.36) μg/mL vs 3.36 (0.59–7.63) μg/mL, *P* = .03). In addition, the adiponectin/leptin ratio was higher in the normal BMI group (145.6 (41.3–495.9) ng/mL) than in the obese group (20.55 (8.74–36.94) ng/mL, *P* = .002).

Compared with the normal BMI T2DM group, the obese T2DM group exhibited a disturbed adipocytokine profile in the form of a significantly increased leptin concentration and reduced adiponectin level. Further studies are needed to determine the causal relationship for this difference and evaluate its importance for personalized diabetic treatment.

## 1 Introduction

Adipose tissue is a key endocrine organ that communicates with brain, muscle, liver, and pancreas, thereby maintaining energy homeostasis. The communication between adipose tissue and other organs is mainly mediated by multiple endocrine substances secreted by adipose tissue, referred to as “adipocytokines.”^[1]^ Changes in the levels of adipocytokines are suspected to be indicators of dysfunction in adipose tissue. Additionally, adipocytokines could provide critical clues regarding the pathophysiological mechanisms of type 2 diabetes mellitus (T2DM).^[2,3]^

Obesity is a common comorbidity of patients with T2DM; therefore, it is important to understand the connection between obesity and T2DM. The results of previous studies suggested that disturbances of adipocytokine secretion may contribute to insulin resistance and/or impairment of insulin production.^[4–6]^ Although relationships between obesity and T2DM have not yet been fully clarified, adipocytokines may play an important role in this interaction.^[7]^

Leptin, resistin, and adiponectin are important adipocytokines that influence both insulin sensitivity and inflammation, which are closely involved in the development of T2DM.^[8]^ Leptin is a proinflammatory molecule that plays a key role in the regulation of glucose and energy homeostasis^[9]^; the results of animal studies have suggested that leptin can normalize hyperglycemia in a manner independent of insulin.^[10]^ Resistin is another proinflammatory cytokine that has been shown to be associated with insulin resistance.^[11]^ Moreover, adiponectin is known to have anti-diabetic, anti-atherogenic, and anti-inflammatory properties. It promotes insulin sensitization by reducing hepatic glucose production and increasing insulin sensitivity in the liver. Thus far, the specific roles of these adipocytokines in human T2DM have not been clearly determined, and their levels appear to vary among study populations.^[12,13]^ This is potentially because the vast majority of studies of adipocytokines in T2DM have been conducted in hospital-based populations, in which the medications used may have significant confounding effects.^[14]^ Therefore, the assessment of adipocytokine profiles in drug-naïve patients who are newly diagnosed with T2DM may help to reveal the relationship between obesity and T2DM; this may aid in personalized hypoglycemic treatment with respect to body mass index (BMI) status. In the present study, we investigated adipocytokine profiles in a cohort of patients who were newly diagnosed with T2DM and who had differing BMIs.

## 2 Methods

### 2.1 Participants

**Figure 1:**
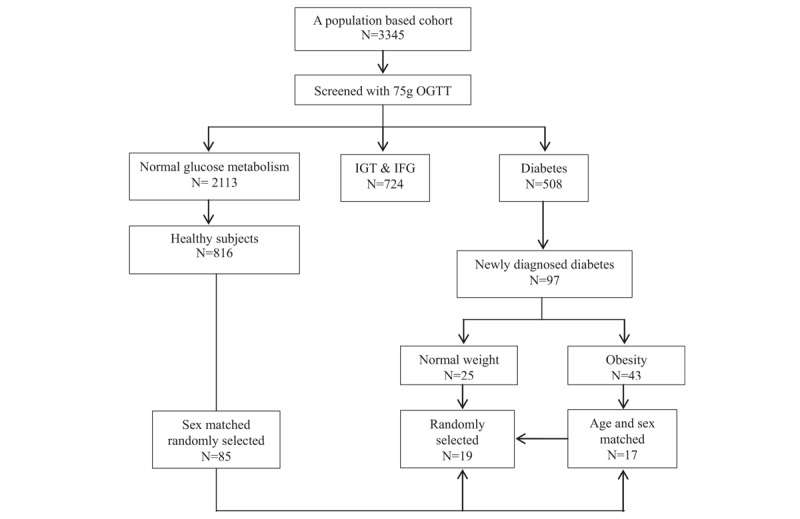
Flow diagram for participant recruitment and sampling. IFG = impaired fasting glucose, IGT = impaired glucose tolerance, OGTT = oral glucose tolerance test.

We conducted a cross-sectional, population-based study of individuals with diabetes and metabolic syndrome in the Pinggu district of Beijing, China between March 2012 and May 2013. All participants completed a 75-g oral glucose tolerance test, unless a clinical diagnosis of diabetes had been made previously. The 1999 World Health Organization criteria for the diagnosis of diabetes were used,^[15]^ and 97 participants were newly diagnosed with T2DM in this manner (they had no known previous clinical diagnosis of diabetes or any previous findings of high blood glucose concentration). Normal body mass was defined as a BMI of 18.5 to <24.0 kg/m^2^ and obesity was defined as a BMI ≥28 kg/m^2^.^[16]^ In total, 19 patients with normal BMI who had been newly diagnosed with T2DM were randomly selected; 17 age- and sex-matched obese patients were also selected from the newly diagnosed diabetic cohort by an independent researcher who was blinded to the laboratory data. The inclusion criteria for the healthy participants were: no history of diabetes (i.e., fasting blood glucose <6.1 mmol/L, 2-hours glucose during a 75-g oral glucose tolerance test <7.8 mmol/L, and glycated hemoglobin <6.0%); BMI <24.0 kg/m^2^; waist circumference <90 cm for men and <85 cm for women; no history of hypertension (i.e., systolic blood pressure <140 mm Hg, and diastolic blood pressure <90 mm Hg); no history of hyperlipidemia (i.e., serum total cholesterol <6.2 mmol/L, triglycerides <1.7 mmol/L, low-density lipoprotein-cholesterol <4.1 mmol/L and high-density lipoprotein-cholesterol ≥0.9 mmol/L for men, high-density lipoprotein-cholesterol ≥1.0 mmol/L for women); normal liver and renal parameters (alanine aminotransferase ≤50 U/L, aspartate aminotransferase ≤40 U/L, and creatinine <104 μmol/L for men and <84 μmol/L for women; no history of hyperuricemia (i.e., blood uric acid <428 μmol/L for men and <357 μmol/L for women); leukocyte count 4 to 10 × 10^9^/L; hemoglobin ≥120 g/L for men and ≥110 g/L for women; no history of smoking; and no use of medication in the preceding 7 days. In total, 85 sex-matched healthy participants were randomly selected (Fig. 1). The institutional review board of Peking University People's Hospital approved the study protocol and written informed consent was obtained from all participants before the study commenced.

### 2.2 Physical examination and sample collection

Sociodemographic information was collected by face-to-face interview. Anthropometric assessments (height, body mass, blood pressure, and waist circumference) were made by trained study staff using standardized procedures. Height and body mass were measured using a pre-calibrated height–mass scale and waist circumference was measured at the mid-point between the lower rib margin and iliac crest. Blood pressure was measured after resting for at least 10 minutes in a seated position; 3 measurements were performed, with 3 minutes intervals between measurements, and the mean value of these 3 measurements was used in subsequent analyses. BMI was calculated as body mass (kg) divided by height squared (m^2^).

Blood samples were obtained after fasting for 8 to 12 hours in the morning. Glycated hemoglobin was measured using high-performance liquid chromatography (Primus Diagnostics, Kansas City, MO). Serum total cholesterol, triglyceride, low-density lipoprotein-cholesterol, high-density lipoprotein-cholesterol, uric acid, aspartate aminotransferase activity, and alanine aminotransferase activity were assessed using enzymatic methods and an automated biochemical analyzer (7170A; Hitachi, Chiyoda, Tokyo, Japan). Plasma glucose and serum insulin were determined by enzyme-linked immunosorbent assay (ELISA) using a biochemical analyzer (7600-120; Hitachi, Tokyo, Japan).

### 2.3 Measurements of adipocytokines

Commercially available ELISA kits were used to quantify adipocytokines, in accordance with the manufacturer's instructions (Millipore, Human Leptin ELISA kit, Cat. # EZHL-80SK; Human High Molecular Weight Adiponectin ELISA kit, Cat. # EZHMWA-64K; Human Resistin ELISA kit, Cat. # EZHR-95K). The intra- and interassay coefficients of variation for leptin, adiponectin, and resistin were 3.5% and 6.5%, 3.2% and 4.4%, and 4.7% and 8.4%, respectively. When calculating adiponectin/leptin and adiponectin/resistin ratios, adiponectin values were converted from μg/mL to ng/mL.

### 2.4 Statistical analysis

The distributions of continuous variables were assessed using the Kolmogorov–Smirnov or Shapiro–Wilk tests. Data were expressed as percentages for categorical variables and means (standard deviations) or medians (interquartile ranges) for continuous variables. Differences between groups were assessed using the chi-squared test or analysis of variance, as appropriate. Binary logistic regression analysis was performed to adjust for BMI and waist circumference when comparing leptin, resistin, and adiponectin concentrations between patients with T2DM who had normal BMI and those who were obese. Differences were considered statistically significant when *P* < .05. Statistical analyses were performed using SPSS for Windows V23.0 (IBM Corp., Armonk, NY).

## 3 Results

Adipocytokine measurements were performed in 121 participants, of which 85 were healthy, while 19 and 17 were patients newly diagnosed with T2DM who had normal BMI and were obese, respectively. The mean BMI of the healthy participants was 21.9 ± 1.4 kg/m^2^, while the mean BMIs for patients with newly diagnosed T2DM who had normal BMI and those who were obese were 22.3 ± 1.4 kg/m^2^ and 31.3 ± 3.0 kg/m^2^, respectively. The mean waist circumference was highest in the obese patients with newly diagnosed T2DM and lowest in the healthy participants, while the value for the patients with newly diagnosed T2DM who had normal BMI was within the range between those 2 values (105.4 ± 7.8 cm vs 75.5 ± 5.3 cm vs 82.4 ± 6.4 cm, respectively; *P *<* *.001). The median glycated hemoglobin concentrations were similar between patients with newly diagnosed T2DM who were obese and those who had normal BMI (*P* = .66). Both the median low-density lipoprotein-cholesterol and median triglyceride concentrations were similar between patients with newly diagnosed T2DM who were obese and those who had normal BMI (*P* = .23 and *P* = .07, respectively). In addition, both median fasting and median 2-hours 75-g oral glucose tolerance test plasma glucose concentrations were similar between patients with newly diagnosed T2DM who were obese and those who had normal BMI (*P* = .43 and *P* = .11, respectively); however, the median serum insulin concentrations were significantly different (6.3 [2.7–8.8] μIU/mL and 21.5 [19.2–24.5) μIU/mL vs 21.1 [12.6–33.0] μIU/mL and 87.8 [56.1–151.6] μIU/mL; *P* < .001 and *P* = .001, respectively) (Table 1).

**Table 1:**
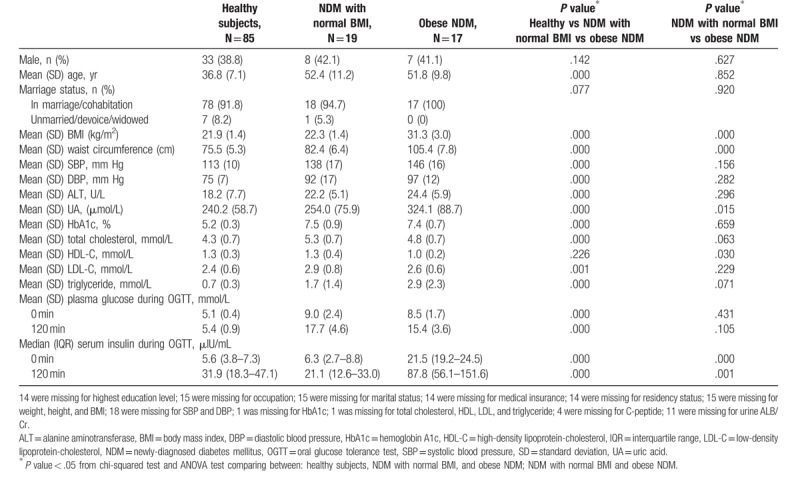
Sociodemographic and clinical characteristics of participants.

### 3.1 Adipocytokine concentrations in healthy participants

**Figure 2:**
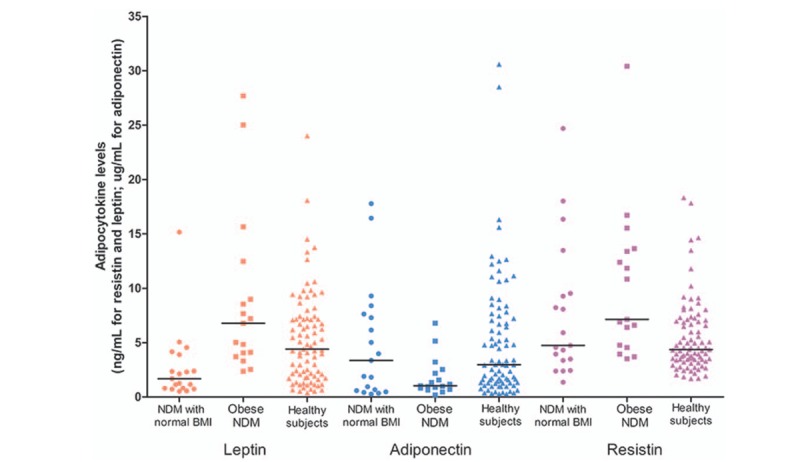
Adipocytokine concentrations in healthy participants and in patients with newly diagnosed T2DM who had normal BMI or were obese. Differences between groups were assessed using the chi-squared test or analysis of variance. BMI = body mass index, NDM = newly diagnosed diabetes mellitus, T2DM = type 2 diabetes mellitus.

In this population-based cohort, we found that the median leptin concentration in the healthy participants was 4.39 (1.86–7.18) ng/mL, whereas the median resistin and adiponectin concentrations were 4.36 (3.41–7.01) ng/mL and 2.96 (1.31–7.33) μg/mL (Table 2 and Fig. 2).

**Table 2:**
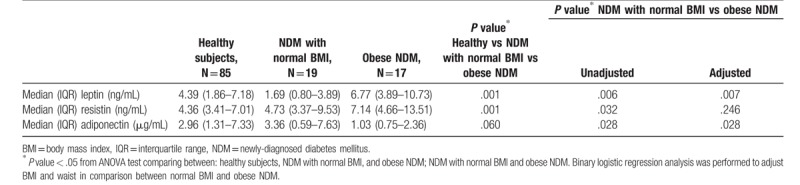
Comparison of adipokine concentrations between different groups.

### 3.2 Adipocytokine concentrations in healthy participants and those with newly diagnosed T2DM

Compared with the healthy participants, the median leptin concentrations were lower in patients with newly diagnosed T2DM who had normal BMI (1.69 (0.80–3.89) ng/mL) and higher in those who were obese (6.77 (3.89–10.73) ng/mL) (*P* = .001). The median resistin concentration was significantly higher in patients with newly diagnosed T2DM who were obese than in either healthy participants or in patients with newly diagnosed T2DM who had normal BMI (7.14 [4.66–13.51] ng/mL, 4.36 [3.41–7.01] ng/mL, and 4.73 [3.37–9.53] ng/mL, respectively; *P* = .001). The differences in median adiponectin concentrations among the 3 groups were not statistically significant (*P* = .06) (Table 2 and Fig. 2).

### 3.3 Adipocytokine concentrations in subgroups of participants with newly diagnosed T2DM

In comparisons between subgroups of participants with newly diagnosed T2DM after adjustment for BMI and waist circumference, the differences in median leptin and adiponectin concentrations remained significant, whereas the difference in median resistin concentration did not (*P* = .007, .03, and .25, respectively) (Table 2 and Fig. 2).

We also compared the median adiponectin/leptin and median adiponectin/resistin ratios between patients with newly diagnosed T2DM who had normal BMI and those who were obese. The median adiponectin/leptin ratio was higher in the normal BMI group (145.6 (41.3–495.9) vs 20.55 (8.74–36.94) ng/mL, *P* = .002), whereas the difference in the median adiponectin/resistin ratio was not statistically significant (*P* = .05) (Table 3).

**Table 3:**
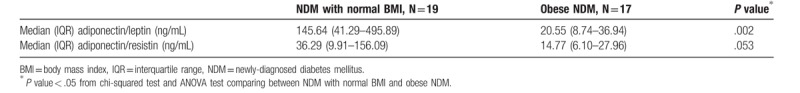
Comparison of adiponectin/leptin and adiponectin/resistin ratios in newly diagnosed diabetes between normal BMI and obese subgroups.

## 4 Discussion

In this study, we have shown that circulating adipocytokine concentrations differed based on the degree of obesity in patients with newly diagnosed T2DM. In a population-based cohort, we demonstrated that higher serum concentrations of leptin and lower serum concentrations of adiponectin were present in patients with newly diagnosed T2DM who were obese than in patients with newly diagnosed T2DM who had normal BMI; moreover, the adiponectin/leptin ratio was also lower in patients who were obese. The disturbed adipocytokine profile presented by patients with T2DM who were obese suggested that obesity and T2DM are related, and that attempts to regulate adipocytokine concentrations could be a promising approach for personalized treatment of patients with T2DM.

T2DM constitutes approximately 90% of all diagnoses of diabetes. Because the diagnosis of T2DM typically involves the initial exclusion of other causes of hyperglycemia,^[17]^ it is not surprising that T2DM is highly heterogeneous with regard to its clinical presentation, progression, and response to treatment. For example, patients with T2DM are generally thought to be obese and insulin-resistant, but not all patients diagnosed with T2DM exhibit this phenotype.^[18]^ Therefore, understanding the relationship between obesity and T2DM would help to clarify the heterogeneity of disease in patients with T2DM and presumably facilitate personalized treatment.^[19]^ Our previous study revealed that a higher level of serum leptin was associated with better glycemic control after 1 year of follow-up^[20]^; therefore, we hypothesized that serum adipocytokine levels may reflect certain pathophysiological defects that differ among patients with T2DM. To better understand the role of adipocytokines in the relationship between obesity and T2DM, we conducted the current study to characterize adipocytokine levels in patients with newly diagnosed T2DM, who exhibited different degrees of obesity.

Adipocytokines are secreted by adipose tissue and serve important roles in energy balance and homeostasis. Leptin, resistin, and adiponectin are closely related to T2DM, as they influence both insulin sensitivity and inflammation. It is commonly believed that leptin and resistin are proinflammatory cytokines, whereas adiponectin has anti-diabetic and anti-inflammatory properties. Although these adipocytokines have been known for many years, their roles in the pathophysiology of T2DM remain controversial.^[7]^ The findings of previous studies have suggested that leptin may represent a predictor of obesity and T2DM^[21]^; however, the serum leptin concentration in patients with T2DM and its associations with other clinical parameters (e.g., BMI, insulin concentration, and waist circumference) remain a matter of debate.^[22,23]^ In addition, comparisons of leptin concentrations between non-obese or overweight patients with diabetes and healthy controls have yielded inconsistent results,^[24,25]^ perhaps due to differences in the methods used for the selection of study participants. The findings of our study showed that serum leptin was lower in patients with newly diagnosed T2DM who had normal BMI than in those who were obese; this difference remained statistically significant after adjustment for BMI and waist circumference. This is consistent with the results of other studies ^[2,26]^ and could be related to a comparative insulin deficiency in the patients with T2DM who had normal BMI.^[27]^

In this study, no significant difference in resistin concentration was identified between patients with T2DM who had normal BMI and those who were obese, following adjustment for BMI and waist circumference. Similarly, Kocot et al did not find any differences in resistin concentration between BMI groups,^[2]^ and another study showed no differences between obese and non-obese patients with diabetes, when compared with non-obese healthy controls.^[28]^ In contrast, Mabrouk et al found that resistin concentrations were higher in obese patients with diabetes than in obese non-diabetic participants; moreover, they were higher in obese patients with diabetes and obese non-diabetic participants than in non-obese healthy controls.^[29]^ This disparity in findings may be due to differences in the study populations; the present study showed that, in a sample of treatment-naïve patients with newly diagnosed T2DM, resistin levels did not differ between individuals who had normal BMI and those who were obese.

Serum adiponectin concentrations have been shown to be inversely correlated with the severity of insulin resistance in patients with T2DM.^[30]^ Consistent with the findings of previous studies,^[18,31,32]^ the present study showed that a lower level of adiponectin was present in patients with newly diagnosed T2DM who were obese than in those who had normal BMI. Furthermore, the difference between these groups remained after adjustment for BMI and waist circumference, which suggests that adiponectin is associated with BMI status in patients with T2DM. Adiponectin is considered to have anti-diabetic and anti-inflammatory effects; therefore, it is reasonable to presume that patients with T2DM who are obese exhibit more severe insulin resistance status than patients with T2DM who have normal BMI.

Previous studies have suggested that adiponectin/leptin and adiponectin/resistin ratios are more closely related to the severity of insulin resistance^[33–36]^; therefore, we calculated these ratios for the patients with newly diagnosed T2DM. Consistent with the findings of the present study, Chearskul et al showed that the adiponectin/leptin ratio was lower in obese adults with T2DM than in non-obese adults with T2DM^[37]^; Kocot et al made similar findings.^[2]^ The adiponectin/resistin ratio has not been extensively characterized in patients with T2DM, prior to the present study; however, the results of a study of patients with gestational diabetes mellitus suggested that this ratio does not differ significantly between patients with early onset gestational diabetes mellitus and those with late-onset gestational diabetes mellitus.^[38]^ Similarly, we did not find a significant difference in this ratio between patients with diabetes who had normal BMI and those who were obese.

Obesity is a frequent comorbidity in patients with T2DM and it has been estimated that at least 90% of these patients are overweight or obese.^[4]^ The risks of many complications and comorbidities (e.g., cardiovascular disease and chronic kidney disease) are considerably increased in patients with T2DM who have concomitant obesity.^[39,40]^ New hypoglycemic therapies, glucagon-like peptide 1 analogue and sodium-glucose cotransporter 2 inhibitor, have been shown to aid in weight loss and to reduce the risks of both cardiovascular disease and chronic kidney disease^[41–43]^; based on this finding, mechanisms linking obesity with T2DM are now an important issue in diabetes research. The results of our study, a comparison of the circulating adipocytokine concentrations between patients with T2DM who are obese and those who have normal BMI, may assist in understanding of the underlying metabolic defects associated with these differing adipocytokine levels.

### 4.1 Limitations

The present study had a few limitations. First, because of the cross-sectional study design, we were unable to infer causal relationships between adipocytokine concentrations and body mass in patients with newly diagnosed T2DM. Second, adipocytokine concentrations are affected by many factors; although we attempted to control for age, sex, BMI, and waist circumference in the present study, a number of other confounding factors might have affected adipocytokine concentrations. To limit the influence of such factors, we recruited a population-based cohort of patients with newly diagnosed T2DM, in which the effects of treatment and comorbidities were presumably limited.

### 4.2 Conclusions

Our findings suggest that, in patients with newly diagnosed T2DM, adipocytokine concentrations (leptin, resistin, and adiponectin) differed between patients who had normal BMI and those who were obese. Patients with T2DM who were obese exhibited a disturbed adipocytokine profile in the form of a significantly increased leptin concentration and reduced adiponectin level, compared with patients with T2DM who had normal BMI. Future studies are needed to identify the causal relationships involved and to determine whether treatment regulating adipocytokine levels could aid in personalized approaches for the management of diabetes.

## Acknowledgments

The authors would like to thank all the participants and researchers involved in this study. The authors would also like to thank Dr. Chong Liu from Peking University for his kind support with data analysis.

## Author contributions

**Conceptualization:** Wei Liu, Xueyao Han.

**Data curation:** Wei Liu, Xianghai Zhou, Yufeng Li, Simin Zhang, Xiaoling Cai, Rui Zhang, Siqian Gong.

**Formal analysis:** Wei Liu.

**Methodology:** Xueyao Han, Linong Ji.

**Writing – original draft:** Wei Liu, Xianghai Zhou, Yufeng Li, Simin Zhang, Xiaoling Cai, Rui Zhang, Siqian Gong.

**Writing – review & editing:** Xueyao Han, Linong Ji.
